# SEXCMD: Development and validation of sex marker sequences for whole-exome/genome and RNA sequencing

**DOI:** 10.1371/journal.pone.0184087

**Published:** 2017-09-08

**Authors:** Seongmun Jeong, Jiwoong Kim, Won Park, Hongmin Jeon, Namshin Kim

**Affiliations:** 1 Personalized Genomic Medicine Research Center, Korea Research Institute of Bioscience and Biotechnology, Daejeon, Korea; 2 Quantitative Biomedical Research Center, Department of Clinical Sciences, University of Texas Southwestern Medical Center, Dallas, TX, United States of America; Natural Resources Canada, CANADA

## Abstract

Over the last decade, a large number of nucleotide sequences have been generated by next-generation sequencing technologies and deposited to public databases. However, most of these datasets do not specify the sex of individuals sampled because researchers typically ignore or hide this information. Male and female genomes in many species have distinctive sex chromosomes, XX/XY and ZW/ZZ, and expression levels of many sex-related genes differ between the sexes. Herein, we describe how to develop sex marker sequences from syntenic regions of sex chromosomes and use them to quickly identify the sex of individuals being analyzed. Array-based technologies routinely use either known sex markers or the B-allele frequency of X or Z chromosomes to deduce the sex of an individual. The same strategy has been used with whole-exome/genome sequence data; however, all reads must be aligned onto a reference genome to determine the B-allele frequency of the X or Z chromosomes. SEXCMD is a pipeline that can extract sex marker sequences from reference sex chromosomes and rapidly identify the sex of individuals from whole-exome/genome and RNA sequencing after training with a known dataset through a simple machine learning approach. The pipeline counts total numbers of hits from sex-specific marker sequences and identifies the sex of the individuals sampled based on the fact that XX/ZZ samples do not have Y or W chromosome hits. We have successfully validated our pipeline with mammalian (*Homo sapiens*; XY) and avian (*Gallus gallus*; ZW) genomes. Typical calculation time when applying SEXCMD to human whole-exome or RNA sequencing datasets is a few minutes, and analyzing human whole-genome datasets takes about 10 minutes. Another important application of SEXCMD is as a quality control measure to avoid mixing samples before bioinformatics analysis. SEXCMD comprises simple Python and R scripts and is freely available at https://github.com/lovemun/SEXCMD.

## Introduction

Next-generation sequencing (NGS) has jump-started a wide array of novel genomics research in the last decade. NGS is replacing two previous technologies—Sanger sequencing and array-based methods. Now, researchers can directly examine genomes, transcriptomes, and epigenomes through whole-exome/genome sequencing, RNA-Seq, DNA methylation, ChIP-Seq, and other methods. In a population-scale experiment, researchers may create hundreds of NGS datasets in a single project, and public databases such as 1000 Genome project [1], Sequence Read Archive (SRA) [2], and The Cancer Genome Atlas (TCGA) [3] have already archived petabytes of information from genome and transcriptome sequencing. However, the sex of individuals analyzed to create these datasets are not always explicitly denoted in the associated reports. Males and females in species with sex chromosomes can have two different combinations of sex chromosomes, XX/XY for mammals and ZW/ZZ for avian, which could lead to different gene expression and DNA methylation patterns. We believe some datasets are assigned the incorrect sex as a result of errors in annotation. Finally, large sequencing centers have sex markers for array-based technology, but not for NGS. If the sex of individuals in a given NGS dataset was known, as part of quality control before bioinformatics analysis, we could prevent errors, including label error, caused by analysis of mixed samples.

Sex-specific genetic markers can be obtained using a simple molecular identification method based on polymerase chain reaction (PCR) [4]. Chaveerach et al. analyzed amplification products from PCR targeting four loci to deduce the sex of individuals sampled [5]. These two methods require primer sequence information *a priori* for a known polymorphic sex marker. Array-based technologies typically use single nucleotide polymorphism (SNP) markers from the human *amelogenin* genes, *AMELX* and *AMELY*, because they are single copy genes and can be used to extract sex-specific sequence markers [6]. Some software packages, such as PLINK [7], PLATO [4], seXY [8], and Golden Helix (Bozeman, MT, USA, http://www.goldenhelix.com), use whole X chromosome heterozygosity or intensities to identify sex. These analyses are based on genotypes, intensities, and heterozygosity thresholds of the X chromosome. Qu et al. took into account both intensities and genotypes of SNPs on the X chromosome simultaneously and calculated the probability of errors in sex identification by logistic regression [9]. The above methods all require SNP polymorphic markers and have biases from copy number variations or large-scale structural variations in individual genomes.

With many pipelines, it is necessary to align NGS reads onto a reference genome in order to identify the genotypic sex of an individual, XX/XY or ZZ/ZW. This process can take hours or days, depending on dataset size, and consume hundreds of gigabytes of disk space. The sex of a given individual can typically be deduced by measuring the B-allele frequency of the X or Z chromosome. Instead of using all NGS reads and the whole reference genome, we developed a simple strategy to use tens of sex-specific marker sequences from syntenic regions of the sex chromosomes. Alignment software chooses whether a given read can be mapped onto one of the sex-specific markers. If the alignment score is not high enough, they are reported as un-mapped. We can thereby collect mapped reads and count the total numbers of hits for each sex-specific marker sequence.

We have implemented our analysis pipeline as (1) designing sex-specific marker sequences, (2) training using a known dataset, and (3) optimal sex marker sequence selection. Because the algorithm uses differences within the sex chromosomes, it can be applied to genome and transcriptome sequencing. Small datasets, such as whole-exome and RNA sequencing, take a few minutes to analyze, while whole-genome sequencing takes more than 10 minutes, depending on computing platform. We have validated our pipeline for two organisms, humans (XX/XY) and chickens (*Gallus gallus*, ZZ/ZW). SEXCMD is freely available at https://github.com/lovemun/SEXCMD.

## Materials and methods

### Extracting sex-specific marker sequences from a reference genome

Sex-specific marker sequences were extracted through six simple steps. First, sex chromosome sequences were aligned with each other using LASTZ [10]. Second, we found a syntenic region between sex chromosomes that did not have long blocks of exactly matching sequences. Blocks without polymorphisms should be less than 35 base pairs (Python script: 2.sex_marker.py)—no longer than read length. This internal parameter can be adjusted if no candidate syntenic regions can be found because the sex chromosome reference genome sequences are incomplete. We used human and chicken reference genomes in this study, and were able to identify appropriate syntenic regions in their sex chromosomes. However, the total number of sex-specific polymorphic regions in the pig genome (susScr3) were not sufficient. Considering recent improvements in long read lengths, i.e., up to 150 bp, minimum length of marker sequences was set to 151 bp with 5 bp mismatches (Python script: 3.sex_marker_filtered.py). This length should be longer than the input NGS read lengths. Finally, we used BLAST [11, 12] to check whether those marker sequences were unique by comparing against all autosomal sequences with minimum identity of 90%. Most of the candidate sequences were removed in this filtering step. If the total number of marker pairs is less than 10, two parameters can be adjusted—–“minimum exact match blocks” in 2.sex_marker.py and “minimum length of marker sequences” in 3.sex_marker_filtered.py. If more than 30 marker sequences are identified, only the sequences within genic regions can be selected to improve efficiency when analyzing data from RNA sequencing. We can assume that most syntenic regions will be within genic regions of sex chromosomes. Non-genic regions can be excluded from the analysis if there are no hits from RNA sequencing alignments. FASTA sequences from final candidate sex-specific marker sequences were indexed by BWA [13] with the “–a is” option. Note that we used the “is” mode because the size of the FASTA file is small.

**Fig 1:**
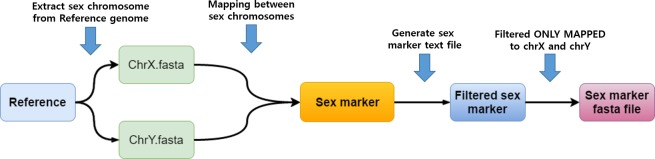
Procedure for extracting sex-specific marker sequences. Two sex chromosomes were aligned with each other using LASTZ, and syntenic regions with polymorphisms were extracted. Final sex-specific marker sequences were selected after removal of similar sequences (90% identity by BLAST).

The workflow for extracting sex-specific marker sequences is depicted in Fig 1. The main goal of this pipeline is to find polymorphic syntenic regions with minimal exact match blocks. Once the sequences of the sex chromosomes are known, we can design sex-specific marker sequences.

Software and URLs are described on the SEXCMD webpage along with examples of command lines.

### Mapping and counting sex-specific hits and interpretation

Each sex-specific marker pair consists of two sex-specific marker sequences originated from the two sex chromosomes. The main concept is to align small numbers of input reads onto those marker sequences and count the number of hits for each pair. Individuals with heterozygous sex chromosomes will have equal numbers of hits on both sex-specific marker sequences in each pair, whereas homozygous individuals will only have hits for one marker sequence per pair (markers on the X or Z chromosome). Because we use only small target sequences instead of alignment with the whole genome, calculation time is reduced. It typically takes hours or days to align all whole-exome/genome or RNA sequencing reads onto a reference genome, but less than tens of minutes to align onto sex-specific marker sequences. We used the BWA-MEM algorithm to align reads onto sex-specific marker sequences and SAMTOOLS [14] to count hits for each marker pair.

Individuals with XY or ZW chromosomes will have a ratio of 1:1 hits for each sex chromosome, and individuals with XX or ZZ chromosomes will have a ratio of 1:0 hits for the two sex chromosomes. Essentially, we used two numbers–the number of hits on the dominant chromosome (Y or W) and the number of hits on the recessive chromosome (X or Z)–to identify the sex of a given individual. Owing to false hits from wrong alignments, we set the minimum fraction of Y or W marker hits on a homozygous individual to be 0.2. If the fraction of hits on the dominant chromosome (Y or W) markers was less than 0.2, we considered the individual to be heterozygous.

The proportion of sex chromosome reads in whole genome sequencing is frequently smaller than in whole exome and RNA sequencing, because whole exome and RNA sequencing are focused on the coding regions of a genome. It takes tens of minutes to align entire datasets onto sex-specific marker sequences. In order to make calculation time shorter, we implemented a ‘test mode’ to measure the minimum number of input reads. It sums the number of hits on each marker and suggests a minimum number of input reads to be analyzed. We recommend that the sum of read counts for all markers be greater than 100 hits. Once we set the minimum requirements, the program uses that number of reads instead of analyzing the entire dataset. This significantly reduces calculation time.

**Fig 2:**
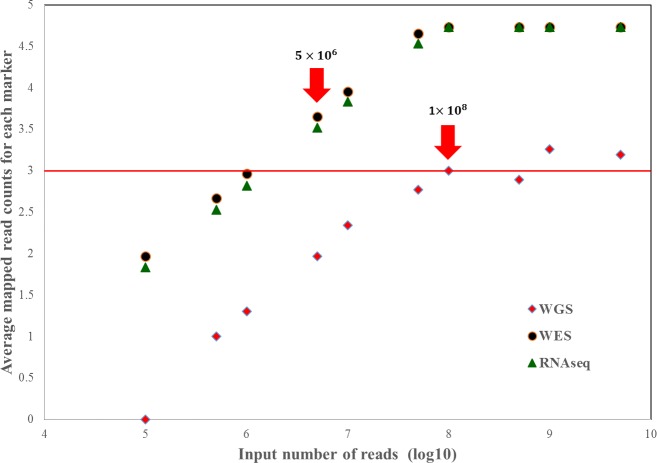
Average read counts for each marker by input number of million sequence reads (log10) for human (hg38) datasets. Red arrows indicate minimum read counts: 5 million (5×10^6^) reads for whole-exome sequencing and RNA sequencing and 100 million (1×10^8^) reads for whole-genome sequencing. The red horizontal line denotes the minimum average read counts of sex-specific marker sequences.

To estimate the minimum number of input reads, we selected a hundred samples for each sequencing type (whole-exome, genome, and RNA sequencing) and measured the average mapped read counts according to input number of reads. The minimum number of input sequence reads necessary for identifying the sex of an individual differs depending on the sequencing type (Fig 2). For human data, we set default values of input read sizes to be five million for whole exome or RNA sequencing datasets and one hundred million for whole genome sequencing datasets.

SEXCMD is a simple and fast method of identifying the sex of given individuals without mapping whole NGS datasets onto a reference genome.

## Results

### Input data

A reference genome with two sex chromosomes is essential for the identification of sex-specific marker sequences. We used human (hg38) and chicken (galGal4) reference genomes from UCSC genome browser (http://genome.ucsc.edu). We downloaded 400 human whole exome sequencing datasets (182 males and 218 females) from the 1000 Genomes Project (http://www.1000genomes.org/data/) and 48 human whole genome sequencing datasets (27 males and 21 females) from the Sequence Read Archive (SRA, http://ncbi.nlm.nih.nih.gov/sra/). In addition, we used 202 human whole exome sequencing datasets and 378 human whole genome sequencing datasets from our in-house database. A total of 131 human RNA sequencing datasets were downloaded (59 males and 72 females) from the ArrayExpress archive (http://www.ebi.ac.uk/arrayexpress/). For chicken, we used 120 whole genome sequencing datasets and 36 chicken RNA sequencing datasets from our in-house database. A summary of these datasets including the sex of individuals sampled is shown in Table 1 and the accession numbers in public databases are provided in S1 Table.

**Table 1:** Summary of datasets used for testing and validation.

Organism	Sequencing type	Male	Female	Total
Human	Whole-genome sequencing	253	163	421
	Exome sequencing	264	338	602
	RNA sequencing	59	72	131
Chicken	Whole-genome sequencing	60	60	120
	RNA sequencing	0	36	36
Total		636	671	1,307

Approximately half of the datasets were from our in-house database and the others were from public databases. Human and chicken were chosen because they have two different configurations of sex chromosomes (XY and WZ).

### Sex-specific marker sequences

Sex-specific marker sequences were derived using Python scripts as described in the Methods section. Genomic coordinates of human and chicken marker sequences are summarized in Tables 2 and 3. In the human genome, SEXCMD selected AMELX, USP9X, NLGN4X, TBL1X, and KDM6A from the X chromosome, and AMELY, USP9Y, UTY, NLGN4Y, TBL1Y, and KDM5C from the Y chromosome. In the chicken genome, SEXCMD selected SMAD2 from the Z chromosome, and FET1 and UBAP2 from the W chromosome. The average length and number of mismatches for marker sequences were 216 bp and 28 in humans and 196 bp and 31 in chickens, respectively.

**Table 2:** Sex-specific marker sequences for humans.

Marker position (chr X)	Gene name	Marker position (chr Y)	Gene name	Seq. len.	Mismatch
chrX:11298648–11298818	AMELX	chrY:6868192–6868362	AMELY	171	14
chrX:11298809–11298973	AMELX	chrY:6868037–6868201	AMELY	165	11
chrX:12975506–12975677	TMSB4X	chrY:13703601–13703772	TMSB4Y	172	23
chrX:41136804–41137021	USP9X	chrY:12726575–12726792	USP9Y	218	18
chrX:41140966–41141217	USP9X	chrY:12735998–12736249	USP9Y	252	29
chrX:41143291–41143443	USP9X	chrY:12738157–12738309	USP9Y	153	15
chrX:41166023–41166214	USP9X	chrY:12773734–12773925	USP9Y	192	19
chrX:41168007–41168218	USP9X	chrY:12776649–12776860	USP9Y	212	28
chrX:41169995–41170234	USP9X	chrY:12778019–12778258	USP9Y	240	25
chrX:41184397–41184675	USP9X	chrY:12786522–12786800	USP9Y	279	36
chrX:41189313–41189475	USP9X	chrY:12793039–12793201	USP9Y	163	22
chrX:41223217–41223402	USP9X	chrY:12846333–12846518	USP9Y	186	15
chrX:45059247–45059466	KDM6A	chrY:13359767–13359986	UTY	220	20
chrX:45060609–45060764	KDM6A	chrY:13358464–13358619	UTY	156	15
chrX:45063422–45063817	KDM6A	chrY:13355003–13355398	UTY	396	73
chrX:45069579–45069955	KDM6A	chrY:13335959–13336335	UTY	377	65
chrX:45069962–45070357	KDM6A	chrY:13335563–13335958	UTY	396	56
chrX:45110079–45110249	KDM6A	chrY:13251017–13251187	UTY	171	19
chrX:45111953–45112110	KDM6A	chrY:13249180–13249337	UTY	158	17
chrX:53193437–53193636	KDM5C	chrY:19706441–19706640	KDM5D	200	15
chrX:53194139–53194577	KDM5C	chrY:19707147–19707585	KDM5D	439	62
chrX:53194546–53194708	KDM5C	chrY:19707554–19707716	KDM5D	163	15
chrX:53195231–53195410	KDM5C	chrY:19708244–19708423	KDM5D	180	15
chrX:53196686–53197044	KDM5C	chrY:19709451–19709809	KDM5D	359	63
chrX:53198490–53198642	KDM5C	chrY:19715352–19715504	KDM5D	153	27
chrX:53198977–53199158	KDM5C	chrY:19715823–19716004	KDM5D	182	15
chrX:53201550–53201743	KDM5C	chrY:19716279–19716472	KDM5D	194	27
chrX:53214689–53214847	KDM5C	chrY:19732584–19732742	KDM5D	159	32
chrX:53217796–53217966	KDM5C	chrY:19741318–19741488	KDM5D	171	20
chrX:53224740–53224908	KDM5C	chrY:19744385–19744553	KDM5D	169	27
chrX:5890658–5890815	NLGN4X	chrY:14843196–14843353	NLGN4Y	158	17
chrX:5890913–5891080	NLGN4X	chrY:14842933–14843100	NLGN4Y	168	19
chrX:5891146–5891303	NLGN4X	chrY:14842706–14842863	NLGN4Y	158	16
chrX:5892286–5892519	NLGN4X	chrY:14841542–14841775	NLGN4Y	234	34
chrX:5892564–5892721	NLGN4X	chrY:14841340–14841497	NLGN4Y	158	23
chrX:6151347–6151560	NLGN4X	chrY:14622026–14622239	NLGN4Y	214	19
chrX:6228566–6228845	NLGN4X	chrY:14522638–14522917	NLGN4Y	280	25
chrX:9465226–9465425	TBL1X	chrY:6910762–6910961	TBL1Y	200	87

Thirty-eight sex-specific marker sequences were generated using the hg38 human genome assembly. Sequence lengths were 158–439 bp with 11–87 mismatches.

**Table 3:** Sex-specific marker sequences for chickens.

Marker position (chr X)	Gene name	Marker position (chr Y)	Gene name	Seq. len.	Mismatch
chrZ:1524420–1524638	SMAD2	chrW:397369–397587		219	39
chrZ:1555791–1555999		chrW:347291–347499		209	50
chrZ:1556251–1556531		chrW:346752–347032		281	54
chrZ:1557294–1557452		chrW:345857–346015		159	14
chrZ:18953921–18954146		chrW:809288–809513		226	52
chrZ:19036774–19036925		chrW:1096613–1096764	FET1	152	5
chrZ:19055988–19056151		chrW:1194410–1194573		164	15
chrZ:435849–436019	ST8SIA3	chrW:513668–513838		171	11
chrZ:437109–437271	ST8SIA3	chrW:519588–519750		163	27
chrZ:439711–439871	ST8SIA3	chrW:523214–523374		161	30
chrZ:53600234–53600410		chrW:619786–619962		177	45
chrZ:7219805–7219996	LOC407092	chrW:132663–132854	UBAP2	192	15
chrZ:7297965–7298239	LOC407092	chrW:98620–98894	UBAP2	275	49
chrZ:7300018–7300231	LOC407092	chrW:97675–97888	UBAP2	214	28
chrZ:7300957–7301146	LOC407092	chrW:96741–96930	UBAP2	190	42

Fifteen sex-specific marker sequences were generated using the galGal5 genome assembly. Sequence lengths were 152–281 bp with 5–54 mismatches.

### Interpretation and identification of sex

We used Python and R scripts to implement SEXCMD. These scripts can be applied to genome and transcriptome sequencing datasets that contain sex-specific fragments. We measured the accuracy of sex identification for humans and chickens using the datasets we downloaded and achieved 100% for all three data types–whole exome, whole genome, and RNA sequencing (Table 4).

**Table 4:** Accuracy of sex identification by SEXCMD.

Source		WGS		WES		RNA-seq	
		Correct/ Total	Accuracy	Correct/ Total	Accuracy	Correct/ Total	Accuracy
**Human**	**Male**	253/253	100%	264/264	100%	59/59	100%
	**Female**	163/163	100%	338/338	100%	72/72	100%
**Chicken**	**Male**	60/60	100%	-	-	-	-
	**Female**	60/60	100%	-	-	36/36	100%
**Total**		536/536	100%	602/602	100%	167/167	100%

SEXCMD showed 100% accuracy of sex identification for human and chicken with all three sequencing data types tested: whole-exome sequencing, whole-genome sequencing, and RNA sequencing.

To obtain accurate results from SEXCMD, we recommend the number of input read sequences should be at least five million. The more input reads used, the better the accuracy of the results. However, calculation time must also be considered. Current implementation of SEXCMD takes approximately 10 minutes for whole genome sequencing, 5 minutes for whole exome sequencing and 1 minute for RNA sequencing datasets.

## Implementation

SEXCMD is available at https://github.com/lovemun/SEXCMD and consists of R and Python scripts. We used a Linux environment for development and validation. Python, R, BWA, and SAMTOOLS should be installed and their PATH included as an environmental variable.

## Conclusions

SEXCMD is a novel pipeline to design sex-specific marker sequences from a reference genome and to identify the sex of an individual analyzed in a given NGS dataset. This pipeline successfully extracts the polymorphic syntenic region of two sex chromosomes, and the NGS dataset of interest will be aligned onto these small sex-specific marker sequences. Then, the number of hits on each sequence is counted. With a few simple criteria, we have achieved 100% accuracies for human and chicken whole exome, whole genome, and RNA sequencing datasets. Perhaps most importantly, the calculation time is typically less than ten minutes because the pipeline only uses a fraction of the input dataset–just enough to identify sex.

This pipeline can be very useful if there is no sex information provided with the NGS reads. Most large genome projects do not give the sex of individuals analyzed because one may think it is trivial information. Even when the sex of individuals is provided, we have found that there is some level of inaccuracy in public databases because of incorrect annotations or mixed samples. Large genome sequencing facilities could use the SEXCMD pipeline prior to bioinformatic analysis to eliminate the possibility of errors due to incorrect annotation or mixed samples.

One limitation of SEXCMD is that it depends on the availability of high quality reference genomes with two sex chromosomes. Many well-studied species have a high quality reference genome, but this is not always the case. SEXCMD may not be able to extract sex-specific marker sequences if the differences between sex chromosomes are not that big enough. If you have a bunch of scaffolds, not in chromosomal level assembly, and if you can decide which the scaffolds are on sex chromosomes of X and Y or Z and W chromosomes, then you can use SEXCMD. One can adjust some parameters in SEXCMD to design proper sex-specific marker sequences. This does not consume CPU time or storage space because SEXCMD is not saving any temporary files.

Most software packages measure the B-allele frequency of X and Z chromosomes and align all input reads onto the reference genome prior to identifying the sex of an individual. For the simple purpose of sex identification, our SEXCMD pipeline is unique.

Finally, we can assume that genotypes of X or Z chromosomes will change if one aligns NGS reads onto heterozygous sex chromosomes regardless of the sex of the given individual. Because the human Y chromosome is inherited from the male parent and is very diverse, researchers tend to ignore this and simply use the XY reference genome for all datasets. This can lead to incorrect genotypes for syntenic regions of sex chromosomes.

We have upload all datasets to SEXCMD github page. Supporting information provides accession number for each sequencing type from 1000 genome project, Sequence Read Archive, and ArrayExpress archive. Because some dataset is ongoing project, the uploaded data contains only mapped sequences to sex markers.

## Supporting information

S1 Table Accession numbers of public database in this analysis. (XLSX)
